# Dataset of trace elements concentrations in snow samples collected in Jelgava City (Latvia) in December 2020

**DOI:** 10.1016/j.dib.2021.107300

**Published:** 2021-09-04

**Authors:** Inga Grinfelde, Jovita Pilecka-Ulcugaceva, Maris Bertins, Arturs Viksna, Vita Rudovica, Sindija Liepa, Juris Burlakovs

**Affiliations:** a Latvia University of Life Sciences and Technologies, Latvia; b University of Latvia, Latvia; c Estonian University of Life Sciences, Estonia

**Keywords:** Air pollution, Atmospheric deposition, Contaminants, Environmental monitoring, Heavy metals, Urban air quality, Snow composition

## Abstract

The data set provided in this article consist of two repeated data sets of chemical elements concentrations in snow samples. The snow samples were collected in Jelgava city at December 15th with 5 day exposition time. Snow samples were collected in 59 monitoring points in Jelgava city and in one sample in rural area monitoring point as control. The collected snow samples were melted, acidified with HNO_3_ and analysed with ICP-MS. The samples were analysed Aluminium (Al), Silicon (Si), Chrome (Cr), Manganese (Mn), Iron (Fe), Nickel (Ni), Copper (Cu), Zinc (Zn), Arsenic (As), Molybdenum (Mo), Cadmium (Cd), Barium (Ba), Tungsten (W), Lead (Pb). The collected data are with fundamental scientific value and can be applied only for local data analysis. Data set is useful for local city air quality research work and for evaluation not only local urban impact but in future evaluate city green infrastructure impact on air quality and evaluation of air pollution mitigation measures efficiency.

## Specifications Table

**Table:** 

Subject	Atmospheric Science
Specific subject area	The urban air quality
Type of data	Table
How data were acquired	Inductively coupled plasma mass spectrometer (ICP-MS). „ Agilent ICP-MS 8900 QQQ” was used for analysis of snow samples.
Data format	Raw; Analyzed
Parameters for data collection	All samples were collected in plastic containers and transported to laboratory.
Description of data collection	One set of chemical elements Al, Si, Cr, Mn, Fe, Ni, Cu, Zn, As, Mo, Cd, Ba, W, Pb in snow samples.
Data source location	Institution: Latvia University of Life Sciences and Technologies City: Jelgava Country: Latvia Latitude and longitude (and GPS coordinates, if possible) for monitoring points are presented in Table S.
Data accessibility	With the article

## Value of the Data

• The urban air pollution is related with increasing human health risks. The knowledge about chemical elements distribution in urban areas helps to develop and improve city infrastructure.
• The data could be very useful for local authorities as well as can be used for fundamental research where urban air pollution issues are investigated.
• The information of air pollution is very useful with temporal distance where by repeating of experiment it is possible to evaluate mitigation measures.
• The collected data can be used to evaluate point-source and nonpoint-source, pollution impact on air quality.
• The multidisciplinary research of dust and chemical elements long distance transport in future will be possible if data in this article are included in models with point source pollution [1] and distribution of chemical elements in catchment areas [2].

## 1 Data Description

The raw data of chemical elements concentrations Al, Si, Cr, Mn, Fe, Ni, Cu, Zn, As, Mo, Cd, Ba, W, Pb in snow samples collected December 2020 are presented in Table S. The unit of concentrations measurement is microgram per liter (µg/l). In Table S first column is ID number of monitoring points. Second and third column represents coordinates of monitoring point. The fourth column represents snow sample number for each monitoring point.

## 2 Experimental Design, Materials and Methods

**Fig. 1:**
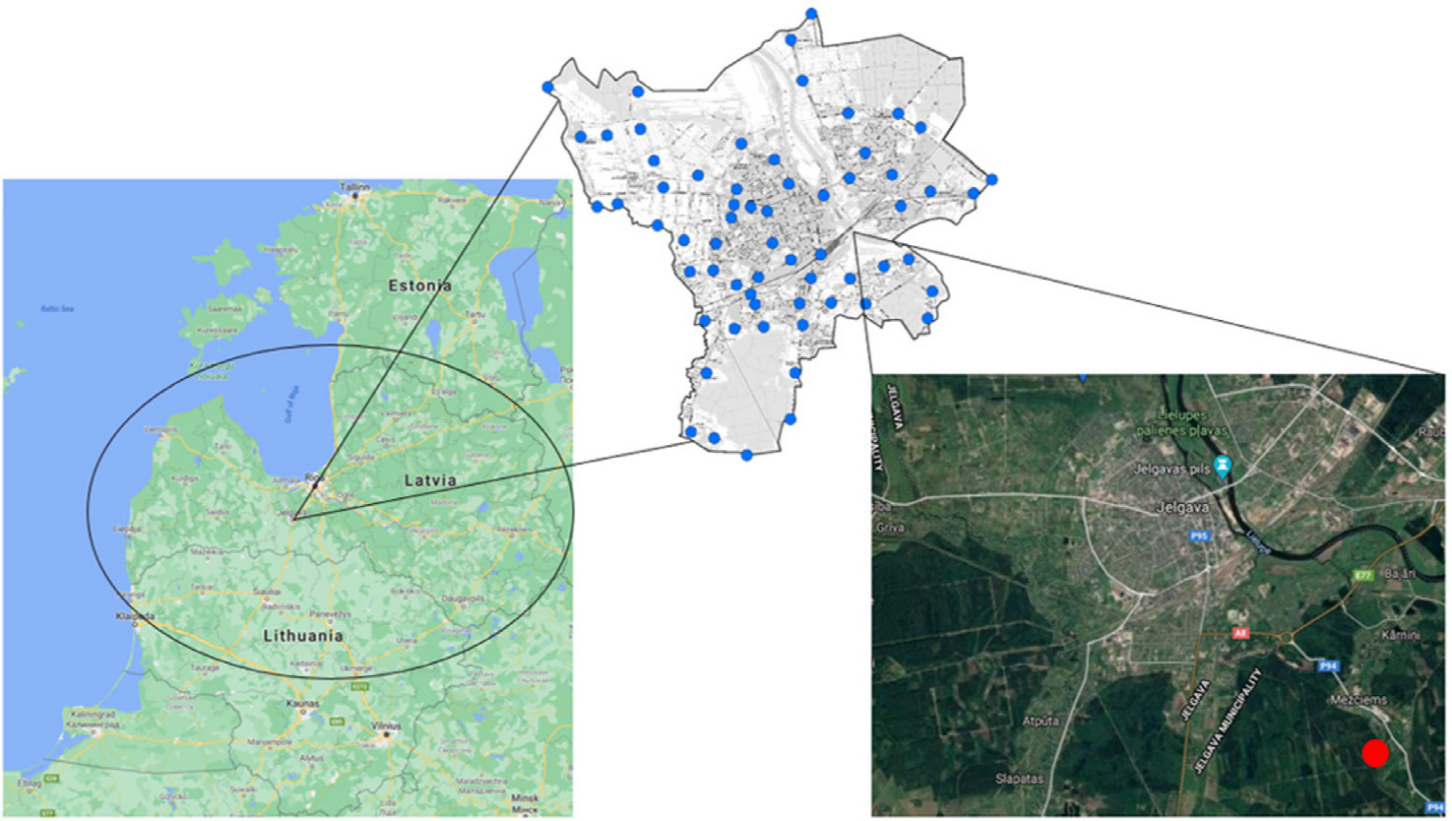
The location of snow sample collection area, the coverage of monitoring points in Jelgava administrative area (blue) and location of rural monitoring point (red).

The location of data collection area is presented in Fig. 1. The Jelgava city with ∼ 57 000 inhabitants is located in central part of Latvia. The sampling areas were selected with aim to monitor transport corridors in Jelgava city. The 59 sampling points were in Jelgava city and one reference point was in rural area at SW from Jelgava city.

**Fig. 2:**
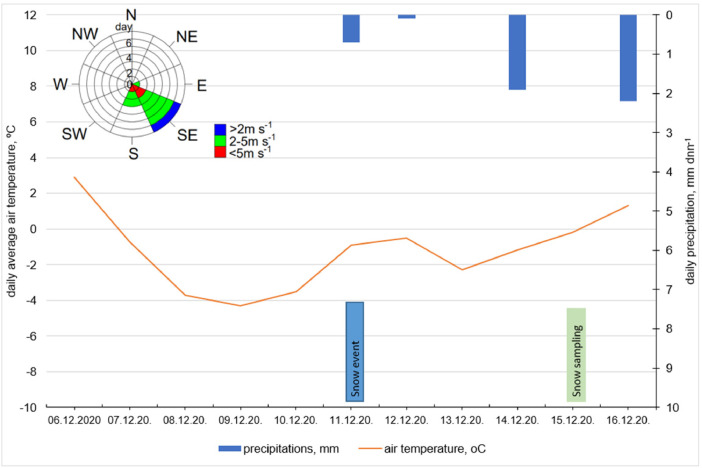
The precipitation and air temperature before sampling period and wind direction and wind speed.

The first snow event in Jelgava was at 11^th^ of December 2020. The snow samples were collected at 15^th^ of December 2020. Snow deposition period was 5 days [4]. This period represent normal city life where transport flow is more active during week days end, less intensive during weekends. The air temperature, precipitations wind direction and wind speed [5] during deposition period is presented in Fig. 2.

**Fig. 3:**
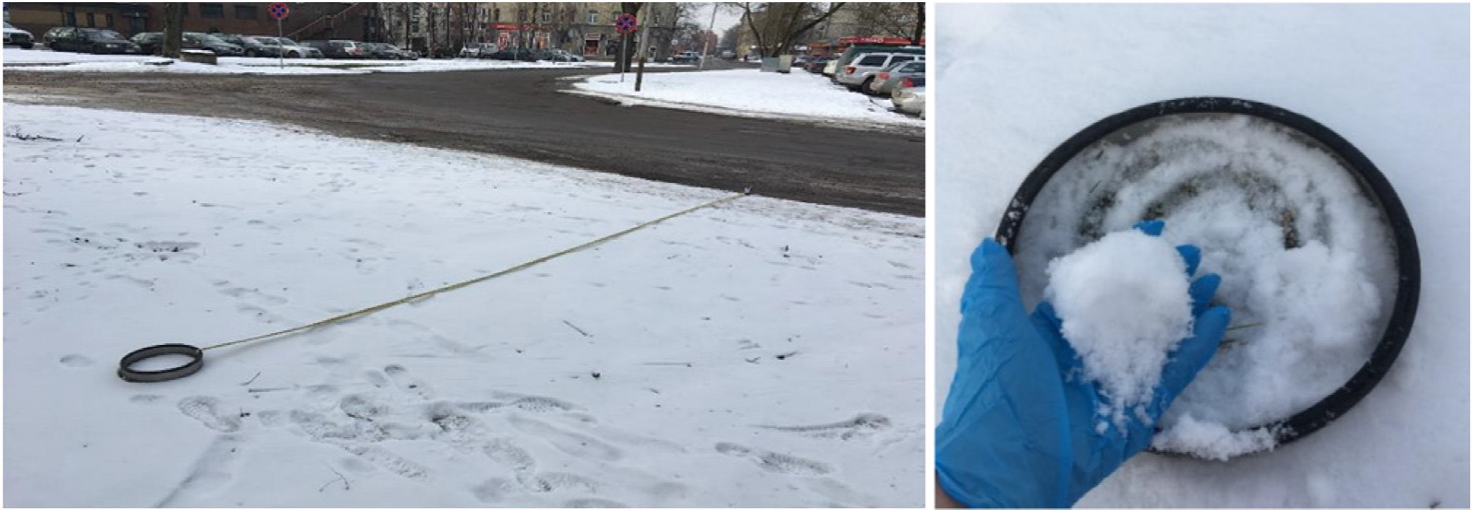
Snow sampling method.

The three snow samples in each monitoring point were collected 5 meters from road [6] edge using measuring tape and 25 cm diameter steel ring covered with teflon Fig. 3. The snow depth was from 7 cm to 19 cm. The snow from ring were collected using disposable dust free nitrile gloves. All snow cover profile from sampling ring were collected in plastic containers and immediately transported to laboratory. The melted snow water was from 378 ml till 436 ml.

In Laboratory snow samples were melted and acidified up to 1% (m/m) HNO_3_ final concentration in solution (1 mL of concentrated HNO_3_, Fischer, TraceMetalGrade 69%, per 150 mL of sample). After 72 h samples were filtered through prewashed (1% (m/m) HNO_3_ water solution) ashless paper filters (Whatman 541) [3], [3], [7], [8], [9], [10]. The Inductively Coupled Plasma Mass Spectrometer “ICP-MS, Agilent 8900 ICP-QQQ” equipped with Micro-mist nebulizer and He collision/reaction cell was used for analysis of chemical elements in snow samples [7], [8], [9], [10]. ICP-MS standard stock solution (10 mg/L, High Purity Standards, ICP-MS-68 A, NIST SRM 3100) was used for the calibration of equipment. Method of external calibration graph with blank correction was used. Deionised water (Millipore, EC < 0.055 μS/cm) was used as blank solution. Calibration graph was constructed in concentration diapason from 0.1 μg/L to 100 μg/L. 10 μg/L internal standard mix solution of Bi, Ge, In, Sc, Tb, Y and Li was used as internal standard for system stability control. One standard solution was introduced into system after every ten samples to verify stability of measurements. Measurements were made in MS/MS configuration using He as collision gas (He flow – 5 mL/min). The instrumental parameters of ICPMS were set as follows: RF power - 1550 W, sampling depth - 8 mm, auxiliary gas flow - 0.90 mL/min, plasma gas flow – 15 L/min.

## CRediT Author Statement

**Inga Grinfelde:** Term, Investigation, Writing - Original Draft; **Jovita Pilecka-Ulcugaceva:** Investigation, Validation, Methodology, Data curation, Writing - Reviewing and Editing; **Maris Bertins:** Formal analysis, Writing-Reviewing and Editing; **Arturs Viksna:** Methodology; **Vita Rudovica:** Writing - Review and Editing; **Sindija Liepa:** Visualisation, Data Curation; **Juris Burlakovs:** Writing - Review and Editing.

## Declaration of Competing Interest

The authors declare that they have no known competing financial interests or personal relationships which have or could be perceived to have influenced the work reported in this article.

## Appendix Supplementary materials

Image, application 1
